# Corrigendum: Translational downregulation of RBCL is operative in the coordinated expression of Rubisco genes in senescent leaves in rice

**DOI:** 10.1093/jxb/ery058

**Published:** 2018-03-10

**Authors:** Yuji Suzuki, Amane Makino

**Affiliations:** 1 Graduate School of Agricultural Science, Tohoku University, Sendai, Japan; 2 CREST, JST, Gobancho, Chiyoda-ku, Tokyo, Japan

Journal of Experimental Botany, Vol. 64, No. 4, pp. 1145–1152, 2013. doi: 10.1093/jxb/ers398

The original version of this article omitted citation information from the figure legends of figures 1, 2, and 4. This information, concerning a study by Suzuki and Makino (2012), has now been added to the online article.

The authors would like to apologise for this error.

